# *In vitro* characterisation of murine pre‐adipose nucleated cells reveals electrophysiological cycles associated with biological clocks

**DOI:** 10.1002/elps.202100308

**Published:** 2022-05-22

**Authors:** Capucine Martin, Jonathan D. Johnston, Erin A. Henslee, Daan R. van der Veen, Fatima H. Labeed

**Affiliations:** ^1^ Chronobiology Section School of Biosciences and Medicine Faculty of Health and Medical Sciences University of Surrey Guildford UK; ^2^ Centre for Biomedical Engineering School of Mechanical Engineering Sciences Faculty of Engineering and Physical Sciences University of Surrey Guildford UK; ^3^ Department of Engineering Wake Forest University Winston‐Salem North Carolina USA

**Keywords:** 3DEP, circadian, dielectrophoresis, electrophysiology, pre‐adipocyte, ultradian

## Abstract

Adipocytes are energy stores of the body which also play a role in physiological regulation and homeostasis through their endocrine activity. Adipocyte circadian clocks drive rhythms in gene expression, and dysregulation of these circadian rhythms associates with pathological conditions such as diabetes. However, although the role of circadian rhythms in adipose cells and related tissues has been studied from phsyiological and molecular perspectives, they have not yet been explored from an electrical perspective. Research into electro‐chronobiology has revealed that electrical properties have important roles in peripheral clock regulation independently of transcription–translation feedback loops. We have used dielectrophoresis to study electrophysiological rhythms in pre‐adipocytes – representing an adipocyte precursor and nucleated cell‐based model, using serum shocking as the cellular method of clock entrainment. The results revealed significant electrophysiological rhythms, culminating in circadian (ca. 24 hourly) cycles in effective membrane capacitance and radius properties, whereas effective membrane conductance was observed to express ultradian (ca. 14 hourly) rhythms. These data shed new light into pre‐adipocyte electrical behaviour and present a potential target for understanding and manipulation of metabolic physiology.

**Abbreviations**
BAT: brown adipose tissue
BRITE: brown‐like adipocytes in WAT'
LD: light–dark
LRRC8a: leucine‐rich repeat containing protein 8a
MEF: mouse embryonic fibroblasts
RBCs: red blood cells
SCN: suprachiasmatic nucleus of the hypothalamus
T2D: type 2 diabetes
TTFL: transcriptional–translational feedback loops
WAT: white adipose tissue

## 1 INTRODUCTION

The mammalian circadian timing system is a complex hierarchical network, organised around an ensemble of uniquely coupled cellular clocks – comprising a principal circadian pacemaker in the suprachiasmatic nucleus (SCN) of the hypothalamus, and autonomous peripheral clocks at a cellular level throughout the body [1]. The SCN is primarily entrained by the environmental, 24 h light–dark cycle, and transmits synchronising cues to the cellular oscillators in tissues throughout the body [2]. Within the diverse cells of both the central pacemaker and the peripheral tissues, the underlying molecular clock mechanism is governed by oscillations in gene expression involving interconnected feedback loops of transcription and translation (transcriptional–translational feedback loops [TTFL]) [3]. There are also other molecular circadian oscillators which can act independently of the TTFL clock [4, 5].

At the level of individual tissue transcriptomes, thousands of genes are brought to exhibit circadian transcription through the actions of the local TTFL‐based cellular oscillators and systemic cues [6]. In this molecular TTFL clock, DNA bindings of heterodimers of CLOCK and BMAL1 cause the transcription of genes which ultimately feedback and inhibit CLOCK‐BMAL1 transcriptional activity. Using integrated luciferase reporters, many cells types, including adipocytes [7], have shown to display these persistent, intrinsically driven, high amplitude rhythms in luciferase with high temporal resolution, allowing longitudinal luminescence recording of rhythmic clock gene expression.

The presence of circadian oscillations in adipose tissues has significant metabolic implications [8, 9, 10]. Therefore, their characterisation may have therapeutic relevance with respect to the pathogenesis and treatment of diseases such as type 2 diabetes (T2D), obesity and metabolic syndrome. Adipose clocks are involved in the regulation of body fat, glucose clearance and insulin production, in the control of the expression of a large array of enzymes implicated in lipid metabolism, and influence adipogenesis, energy as well as vascular homeostasis [11, 12, 13]. White adipose tissue (WAT) has long been recognized as the main site for excess energy storage [14]. Disruption of circadian timing of adipose clocks occurs when food intake, activity and sleep are altered [15], and understanding how these many tissue clocks are synchronised to tick at the same time each day and determining how each tissue ‘senses time’ set by these molecular clocks might open new insights into human disease, including sleep disorders, circadian disruptions, diabetes and obesity. The importance of adipose tissue in the development of obesity and diabetes linked with circadian desynchrony makes this tissue of great interest.

In addition to gene expression, variations in intracellular ion levels (in particular K^+^) have been associated with the endogenous clock mechanism in cells such as neurons, implicating the membrane potential as an intrinsic part of the clock [16]. However, the membrane potential is difficult to measure directly; the gold‐standard ‘patch clamp’ technique is very slow and cumbersome, typically only allowing the analysis of a few cells per day, and measurement of individual cells takes too long to reliably measure circadian variation. An alternative method involves the measurement of the passive electrical properties of cells using dielectrophoresis (DEP) [17], a cellular analogue to electrophoresis. Unlike electrophoresis which uses a DC voltage to measure charge, DEP can use AC signals over a range of frequencies to determine properties such as the effective membrane conductance and capacitance and cytoplasm conductivity (*σ*_cyto_), which affect the response in different frequency ranges [17]. DEP has been used to study the circadian clock in red blood cells (RBCs) which are anucleate and lack the TTFL circadian clock found in (almost all) nucleated mammalian cells. Following pharmacological and ion replacement interventions [18], human RBCs exhibited circadian rhythms in *G*_eff_ (effective membrane conductance) and *σ*_cyto_ that were found to be regulated by potassium (K^+^); this was the first study to link DEP technology with the field of chronobiology. Using DEP, rhythmic electrophysiological results were also reported in vole RBCs (which typically oscillate on a much shorter timescale), further enforcing the versatility of this system in both species and temporal resolution [19].

Whilst adipose cells and related tissues are widely studied using conventional molecular methods, they have been studied infrequently from an electrical perspective. As our research into electro‐chronobiology has revealed electrical properties having important roles in TTFL‐independent RBCs, we have here used DEP to study electrophysiological rhythms in 3T3‐L1 pre‐adipocyte cells, a robust adipocyte precursor and nucleated cell‐based model used in adipocyte research. We have previously demonstrated a TTFL clock in this cell model [20]; however, it is unknown as to whether an ionic component exists in the TTFL clock mechanism. If such a role were found to exist, it opens new avenues to understanding the regulatory physiology of such cells, and the way in which deviation from this rhythm might affect the function of both cell and wider organism.

Adipogenesis is the process by which multipotent precursor mesenchymal stem cells differentiate into lipid–laden adipocytes [21], traditionally classified as either WAT or brown adipose tissue (BAT), respectively, or brown‐like cells within white adipose depots which are known as beige (or BRITE) adipocytes [22, 23, 24]. WAT and BAT are involved in energy balance [25, 26], and adipocytes have been linked to obesity related studies, particularly as obesity is seen as a serious public health problem. Adipose tissue was also found to facilitate, through different physiological pathways, calorie storage and glucose homeostasis [26]. This process is regulated by a complex and highly orchestrated gene expression programme involving a cascade of transcription factors that together lead to the establishment of the differentiated adipocyte state [21, 27].

Although the ionic clock mechanism present in RBCs is required as part of a non‐transcriptional circadian rhythm in RBCs, it is unclear whether an ionic component is present in nucleated cells which use transcriptional clocks to operate. In this paper, we test the hypothesis that rhythmic behaviour is present in both the electrical properties and molecular markers of pre‐adipocyte cells, and that electrical parameters can be used as a marker for biological rhythms in nucleated cells. Our results indicate that pre‐adipocytes express endogenous electrophysiological rhythms; circadian rhythms were observed in *C*_eff_ (effective membrane capacitance) and cell radii properties, and strikingly *G*_eff_ exhibited an ultradian rhythm. We believe that these DEP data in TTFL expressing pre‐adipocytes reveal novel pre‐adipocyte electrophysiology and that present a potential target for understanding and manipulating metabolic physiology.

## 2 MATERIALS AND METHODS

### 2.1 3T3‐L1 *Per2*‐d*Luc* pre‐adipocytes cell culture

The cells were kindly donated by the team of Dr Andrew C. Liu from the University of Memphis, USA. The cells contain an integrated lentiviral luciferase reporter of either the *Per2* or the *Bmal1* gene promoter. A mammalian reporter construct contains an expression cassette in which the promoter of a circadian gene (in this case *Per2* and *Bmal1*) is fused with the *Luciferase* gene [28]. The lentiviral reporters were generated by a recombination‐based Gateway cloning method.

Murine pre‐adipocytes were grown in Dulbecco's Modified Eagle's Medium (DMEM, Sigma‐Aldrich – D6429) and kept in an incubator at 37°C (95% humidified air and 5% carbon dioxide, CO_2_). The medium contained a high glucose concentration of 4500 mg/L, supplemented with 10% of foetal bovine serum (FBS, Gibco – 16000‐036), 2 mM l‐glutamine (Gibco – 25030‐081), 1% antibiotic–antimycotic (Thermo Fisher Scientific – 15240‐062) and 1% sodium pyruvate (Sigma‐Aldrich – S8636). The antibiotic–antimycotic mixture contained 10000 units of penicillin, 10 mg of streptomycin and 25 µg of amphotericin B. Cells were grown to reach approximately 80% confluence before seeding. For seeding, the culture medium was aspirated and cells were washed with 1×‐phosphate buffered saline (PBS, Sigma‐Aldrich – P4417) twice. After washing, 2 ml of 1× trypsin–ethylenediaminetetraacetic acid (Trypsin–EDTA, Sigma‐Aldrich – T4172) was added to the cells before incubation at 37°C, 5% CO_2_, for approximately 2 min or until all the cells were detached from the flask. Upon full cell detachment, the trypsin–EDTA was inactivated by an addition of 2 ml of fresh culture medium to the flask, and the resulting suspension was gently mixed. The cells were centrifuged at 200 *g* for 4 min. After the removal of the supernatant, the cell pellet was then resuspended in fresh culture medium for continual growth, or in the appropriate media required for subsequent analysis, with the cell concentration adjusted accordingly.

### 2.2 Cell entrainment by ‘serum shock’

The molecular clocks within individual cells can be synchronised by using high serum concentrations [29, 30] – a well‐established protocol for TTFL synchronisation between cells, often referred to as ‘serum shock’ or ‘serum pulse’ – which has been developed in 1998 [31]. The ‘serum shock’ medium was prepared by mixing 50% culture medium (DMEM) and 50% FBS, supplemented with 1% of antibiotic–antimycotic. To serum shock, the cells were grown to 90% confluence. The culture medium was removed as described earlier before cells were washed with 1× PBS. The 50% serum containing medium was then added to the cells (for example, 2 ml in a 35 mm dish, 6‐well plate, or 50 µl in 96‐well plates), and the cells were incubated at 37°C and 5% CO_2_ for a total of 2 h, after which cells were returned to normal serum conditions.

### 2.3 Verification of circadian gene expression, using bioluminescence recording

Bioluminescence reporting of clock gene expression is the gold‐standard method of measuring circadian clock rhythms in nucleated cells. Real‐time bioluminescence signals reporting the activity of the *Per2* and *Bmal1* promoter were measured using luciferin – a substrate of the luciferase enzyme. For bioluminescence, cells were kept in DMEM that is free of phenol red (Gibco – 21063‐029), to avoid potential dye interference with the bioluminescence recordings. This medium was also supplemented with 10% FBS, 1% antibiotic–antimycotic, 1% sodium pyruvate and 0.1 mM luciferin (Promega E6551, Southampton, UK). After 2 h of 50% serum‐containing medium, cells were first washed with 1×‐PBS prior to adding the bioluminescence recording medium (at an approximate volume of 2 ml per 35 mm diameter dish). Finally, the dishes were sealed using a 40 mm diameter cover glass (VWR, Poole, UK) sealed to the dish using a translucent grease (ACC Silicones SGM494, Somerset, UK). Bioluminescence was recorded using a luminometer device constructed for this purpose (version 2.31, Actimetrics, UK). This machine was a light–tight box containing 4 photomultiplier tubes and a turntable with 32 slots for the 35 mm culture dishes. The real‐time bioluminescence of each dish was measured for approximately 70 s, at intervals of 10 min. Bioluminescence rhythms were usually recorded for 3–10 days. Due to high transient luminescence upon medium change, the first 24 h of recordings were excluded for rhythm analysis [7]. The analysis programme allowed the export of baseline‐subtracted data calculated on a 24 h running window, to which a cosine curve was fitted.

### 2.4 DEP

DEP was used to determine the impedance properties of the cells by measuring the changes in observed DEP behaviour across a range of frequencies [17], and fitting a model to the data to estimate the properties [32, 33, 34]. Cells were analysed in a low‐conductivity DEP medium prepared using an iso‐osmotic (285 mOsm/kg) solution comprising 250 mM sucrose (Sigma‐Aldrich, S8501) and 17 mM glucose (Sigma‐Aldrich, G8270) supplemented with 0.1 mM calcium chloride (CaCl_2_, Sigma‐Aldrich – C1016) and 0.25 mM magnesium chloride (MgCl_2_, Sigma‐Aldrich – M8266) in Milli‐*Q* water [35]. The medium conductivity was adjusted to 55 mS/m using 1× PBS and verified with a conductivity meter (Hanna instruments, Wolflabs, York, UK). Cells were washed twice before centrifugation at 200 *g* for 4 min, followed by resuspension in 5 ml of DEP medium adjusted to a cell concentration of 1 million cells/ml. A volume of 75 µl cell suspension was added to a 3DEP chip and inserted into a 3DEP reader (Deptech, Heathfield, UK). Wells were energised 10 V_p–p_ for 30 s with a frequency range of 10 kHz 45 MHz. DEP measurements were performed every 4 h between 0 and 48 h following serum shock of the cells. A total of 7–9 technical repeats per sample were recorded. The biological replicates of each time course (*n*) were 5 for the first 0–20 h following serum shock, and *n* = 6 for 24–48 h. The 3DEP system optically analyses the redistribution of cells due to DEP across 20 frequencies [32]; the 3DEP analysis software [19] then fits a single‐shell model (comprising a membrane ‘shell’ and cytoplasm ‘core’) [33, 34] using the 3DEP software's built‐in Levenberg–Marquardt curve‐fitting algorithm [19]. Equations and full description of fitting to determine cellular properties was as published previously. The cell model was used to generate the cytoplasm conductivity *σ*_cyto_ (S/m), effective membrane conductance *G*_eff_ (S/m^2^), and effective membrane capacitance *C*_eff_ (F/m^2^). The medium conductivity (S/m) and cell radii measurements were manually inserted into the software and the medium relative permittivity (*ε*) was fixed at 78 [18]. For cell radius measurements, 10 µl of the final 1 ml cell suspension in DEP medium (at the approximate concentration of 1 × 10^6^ cells/ml) was pipetted into a disposable C‐Chip haemocytometer (Labtech, Heathfield, UK), and pictures of the cell suspensions were taken before each of the DEP recordings using a microscope (Nikon TMS‐F) adapted to a digital camera (Nikon, COOLPIX990) and via the Dolphin Imaging software version 8.0. For each sample, the diameter of 50 different cells was determined using ImageJ version 2.0 and averaged before the value was inserted into the software prior to analysis. Images were taken from a total of three biological replicates.

### 2.5 Statistics

For each time point, parameters obtained by DEP and bioluminescence were plotted across circadian time, each biological replicate considered, and both a straight line + cosine curve was fitted to the data, and an extra‐sum‐of‐squares was used to test the hypothesis that this model was a better fit than a straight line, using *α* = 0.05. All graphs were made using Sigma Plot version 12.

## 3 RESULTS

### 3.1 Verification of clock genes *Per2* and *Bmal1*‐driven circadian cycles in pre‐adipocytes

**FIGURE 1:**
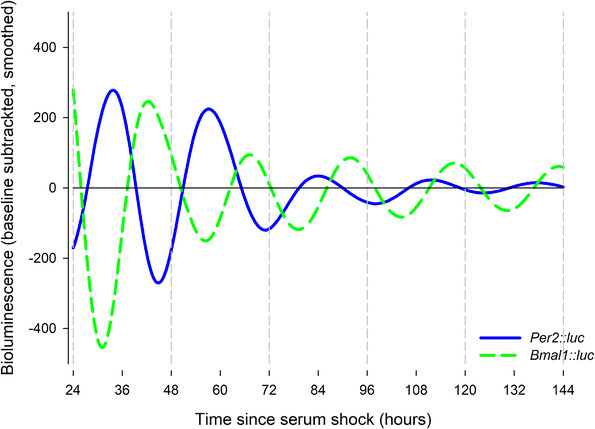
Bioluminescence traces showing *Per2* and *Bmal1* rhythms. *Per2* and *Bmal1*‐d*Luc* pre‐adipocytes display anti‐phasic bioluminescence rhythms. Data are presented as baseline‐subtracted data of bioluminescence for both reporters, for *Per2‐*d*Luc* (blue) and *Bmal1‐*d*Luc* (green)

3T3‐L1 *Per2‐*d*Luc* and *Bmal1*‐d*Luc* pre‐adipocytes reporter cells were directly used in bioluminescence recording. These cells displayed persistent bioluminescence rhythms in 35 mm culture dishes monitored in a LumiCycle luminometer. *Per2*‐d*Luc* and *Bmal1*‐d*Luc* reporters displayed anti‐phasic rhythms of bioluminescence, consistent with their reported anti‐phasic expression in the TTFL as a function of E‐box‐ and RORE‐containing promoters in regulating distinct and opposite phases of gene expression (Figure 1).

### 3.2 Pre‐adipocytes exhibit circadian rhythms in cell radius and *C*_eff_ measurements

**FIGURE 2:**
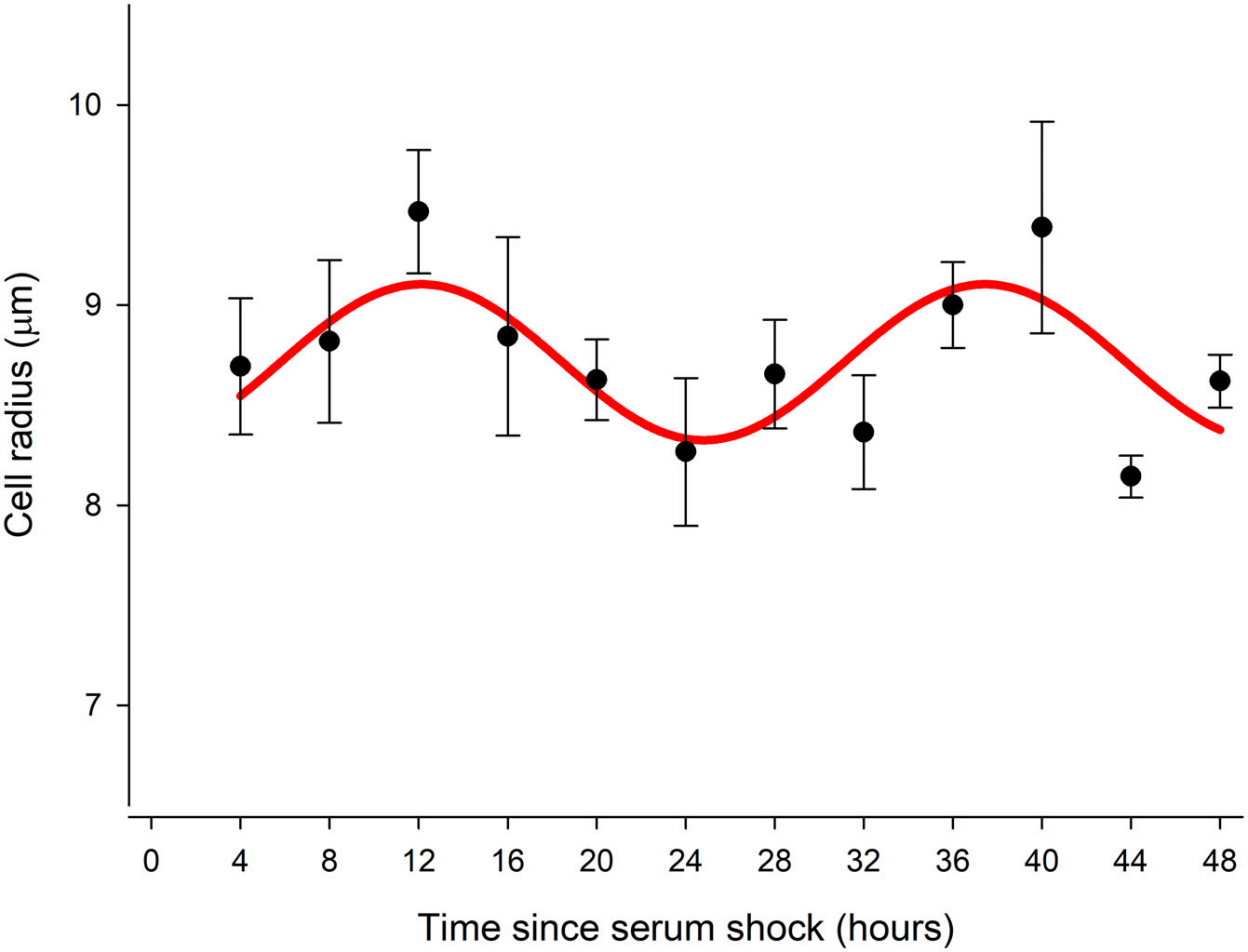
Circadian rhythms were observed in cell radii measurements of pre‐adipocytes after serum shock. Cell radii averaged values across the circadian time. Cell radius is presented as mean ± SEM for each time points. A total of *n* = 5 biological replicates between 4 and 20 h following serum shock and *n* = 6 biological replicates between 24 and 48 h after serum shock are presented. Each biological replicate consists of the mean radius of 50 cells measured in that sample. Cosinor analysis (red line) reveals a significant circadian rhythm (*p* < 0.05), with a period of 26:19 h

**FIGURE 3:**
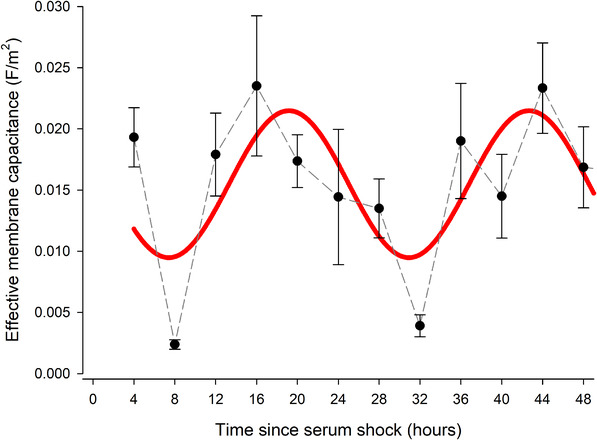
Circadian rhythms were observed in pre‐adipocyte *C*_eff_ values after serum shock. *C*_eff_ of 3T3‐L1 *Per2*‐d*Luc* pre‐adipocytes recorded using DEP across the circadian time. Membrane capacitance is presented as mean ± SEM for each time point, connected by a dashed grey line. A total of *n* = 5 biological replicates between 4 and 20 h following serum shock and *n* = 6 biological replicates between 24 and 48 h after serum shock are presented. Cosinor analysis (red line) reveals a significant circadian rhythm (*p* < 0.005), with a period of 23:30 h. DEP, dielectrophoresis

Both cell radius and capacitance exhibited rhythmic behaviour with circadian periodicity of 23.3 h (*p* < 0.005) and 26.2 h (*p* < 0.05), respectively (Figures 2 and 3 and Table 1). This contrasts with circadian rhythms in non TTFL containing human RBCs, in which both of these parameters were arhythmic [18]. Of particular interest is that two rhythms exhibited a ca. 6 h phase difference, whereas one explanation for a change in capacitance might be a cell swelling causing the membrane to stretch, which would place the two measures in antiphase. A 90° phase shift suggests that their relationship is correlative and may have a common cause but is not directly causative.

**TABLE 1:** A summary of pre‐adipocyte rhythmic properties (with cosine as a preferred fit relative to a straight line)

	***p*‐Value**	**Period (h)**	**Acrophase (h)**
Cell radii (µm)	<0.05	26:19	13:27
*C*_eff_ (S/m^2^)	<0.005	23:30	19:10
*G*_eff_ (S/m)	<0.0001	14:35	15:11
*σ*_cyto_ (S/m)	N.S.		

*Note*: A summary of periodicity and acrophase (or peak time) following serum shock in: cell radii, *C*_eff_, *G*_eff_ and *σ*_cyto_. All parameters, with the exception of *σ*_cyto_, demonstrated significant temporal variation (*p* < 0.05), with a cosine curve being the preferred model (vs. a straight line).

### 3.3 Pre‐adipocytes exhibit ultradian rhythms in *G*_eff_ but no significant rhythms in *σ*_cyto_

**FIGURE 4:**
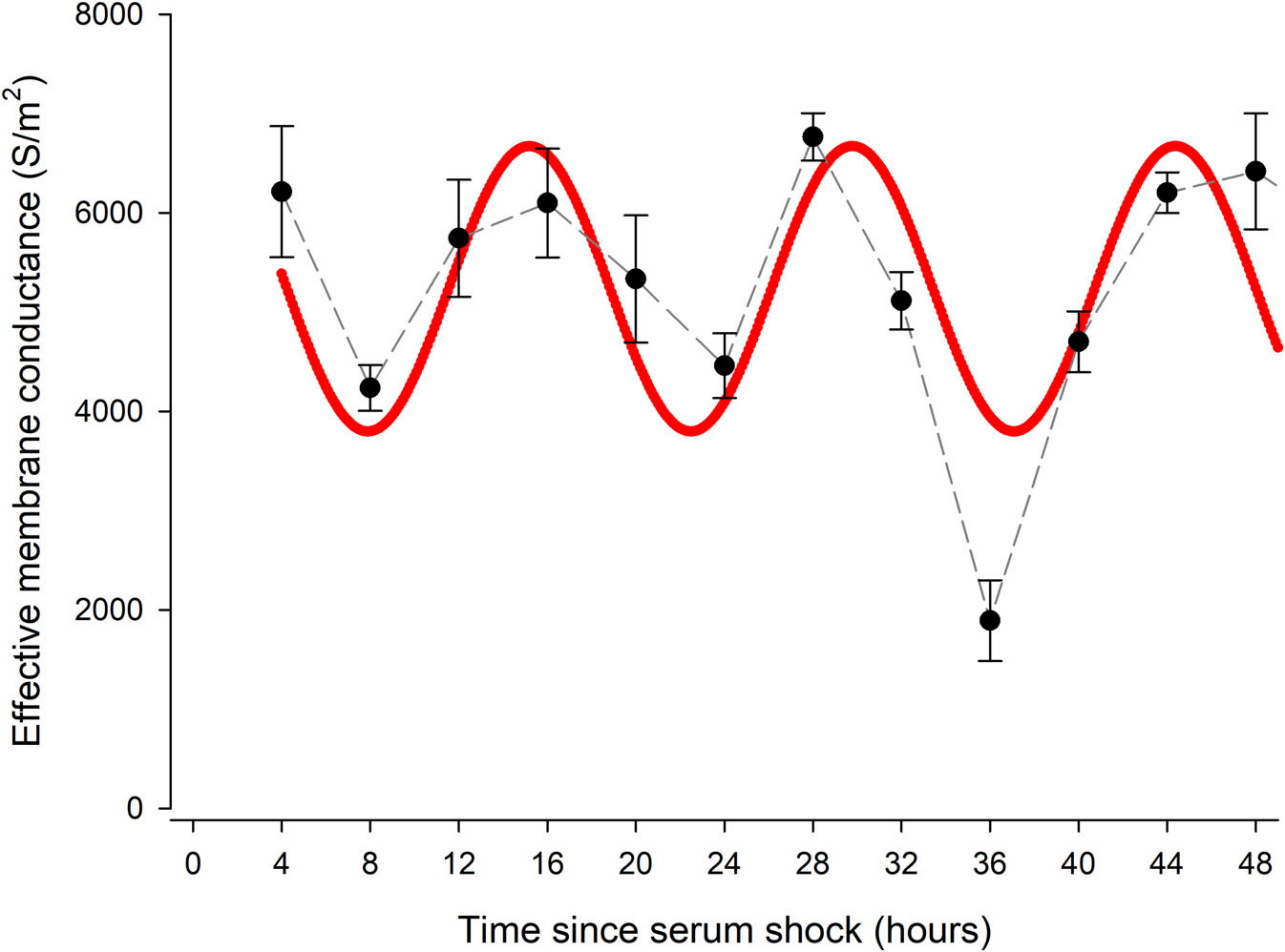
Ultradian rhythms were observed in pre‐adipocyte *G*_eff_ values after serum shock. *G*_eff_ of 3T3‐L1 *Per2*‐d*Luc* pre‐adipocytes recorded using DEP across the circadian time. Values are presented as mean ± SEM for each time point, connected by a dashed grey line. A total of *n* = 5 biological replicates between 4 and 20 h following serum shock and *n* = 6 biological replicates between 24 and 48 h after serum shock are presented. Cosinor analysis (red line) reveals a significant ultradian rhythm (*p* < 0.0001), with a period of 14:35 h. DEP, dielectrophoresis

**FIGURE 5:**
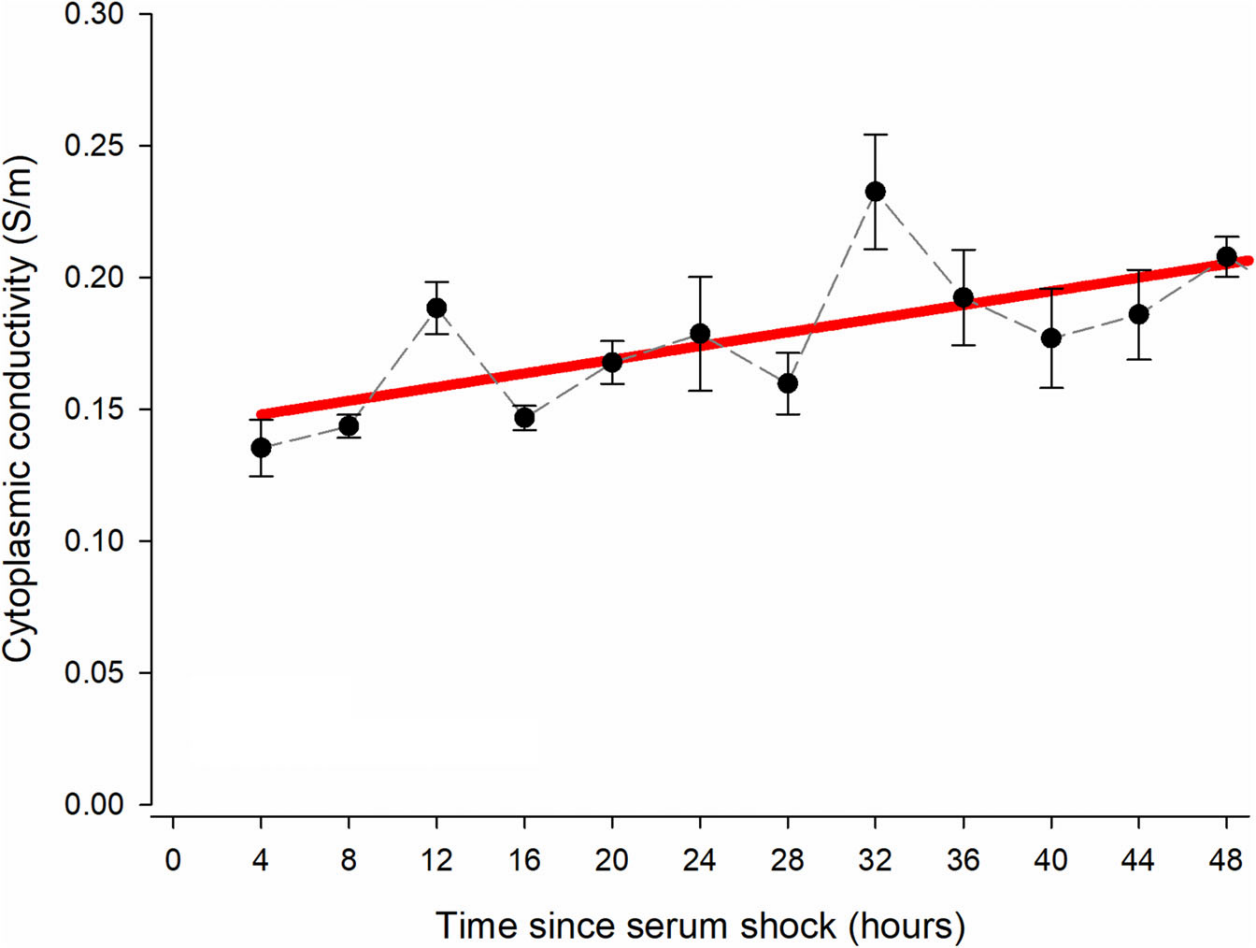
No significant rhythms were observed in pre‐adipocyte *σ*_cyto_ values after serum shock. *σ*_cyto_ of 3T3‐L1 *Per2*‐d*Luc* pre‐adipocytes recorded using DEP across the circadian time. Values are presented as mean ± SEM for each time point, connected by a dashed black line. A total of *n* = 5 biological replicates between 4 and 20 h following serum shock and *n* = 6 biological replicates between 24 and 48 h after serum shock are presented. Cosinor analysis (red line) reveals no significant rhythms were seen (*p* > 0.05). DEP, dielectrophoresis

The membrane conductance parameter, *G*_eff,_ also exhibited a strong rhythmic variation (*p* < 0.0001). However, intriguingly, the period was shorter than those observed for *C*_eff_ and radius, with a cosine best fit period of 14.4 h (Figure 4 and Table 1). This suggests that the *G*_eff_ rhythm may be ultradian rather than circadian. It is however contrary to the *G*_eff_ oscillator in human RBCs, which exhibited a ∼24‐h rhythm, suggesting a different underlying clock mechanism [18]. As RBCs are anucleate, the RBC clock mechanism is not based on expressions of *Per2*, *Bmal1*, *Clock* and so on, suggesting that although the clock mechanisms in both nucleate and anucleate cells involve ion trafficking, they do so in ways that form different parts of their respective clocks. Interestingly, and again unlike RBCs, no statistically significant rhythmic behaviour was identified in *σ*_cyto_ (Figure 5).

## 4 DISCUSSION

This study represents the first observation of rhythmic behaviour of electrical properties in nucleated cells employing a TTFL clock. It demonstrates that circadian mechanisms at least alter cell electrophysiology, though at this point it is unclear as to whether the observed changes actually form part of the clock itself or are epiphenomena of the existing molecular clock mechanism.

**FIGURE 6:**
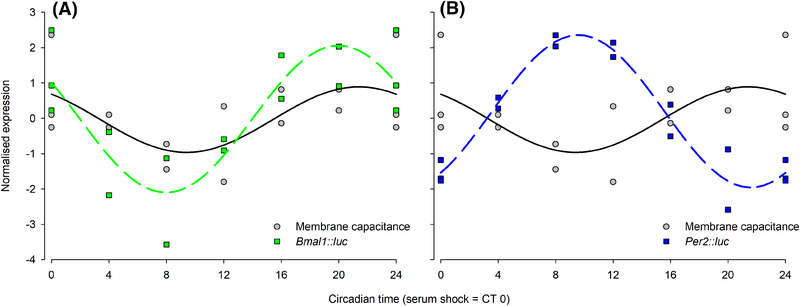
Rhyhmic relationships between *Per2/Bmal1* and *C*_eff_. Normalized values (data were *z*‐scored on a circular scale) of (A) *C*_eff_ and *Bmal1* and (B) *C*_eff_ and *Per2*, together with best fit calculated cosine fits to the data. Data are plotted over one circadian period. *C*_eff_ and *Bmal1* exhibit similar rhythms in co‐phase, whereas *C*_eff_ and *Per2* exhibit similar rhythms in anti‐phase

In order to better understand the relationship between the electrical clock and the TTFL clock, we compared the DEP‐derived results to those taken from the gold‐standard bioluminescence test used in TTFL monitoring. When the observed rhythms in *C*_eff_ and *Per2* and *Bmal1‐*driven bioluminescence were compared, a close co‐phasic relationship was observed between *C*_eff_ and *Bmal1*; and a close *anti*‐phasic relationship between *C*_eff_ and *Per2* (Figure 6). Although this does not infer a causal relationship – the transcriptional pathways of both genes are sufficiently complex to prohibit identification without further analysis – it does indicate that *C*_eff_ provides a clear non‐invasive internal circadian phase marker. This extends our previous demonstration of DEP as a phase marker for circadian rhythms in RBCs from humans [18, 36, 37] and voles [19] to now demonstrate that DEP‐measurable rhythms are *also* present in nucleated pre‐adipocytes, in contrast with results determined in a detailed study of biological rhythm behaviour via DEP in human RBCs [38, 39]. Our pre‐adipocytes exhibited circadian changes in both radius and capacitance, whilst not demonstrating a rhythm in *σ*_cyto_.

Usually, *C*_eff_ is indicative of cell size or membrane morphology; as cells swell, surface features are smoothed out, meaning that cell capacitance is typically inversely related to cell radius [40]. However, our data suggest that the circadian rhythm in *C*_eff_ is associated with a 90^°^ phase shift in capacitance relative to cell size. The cyclic changes in cell size may be associated with anticipated physiological changes to maintain osmostasis. The cyclic *C*_eff_ may be driven by changes in morphology or composition as much as by changes in stretching or increase in relative surface area. It could also be linked to chemical compounds located at the membrane, including cell surface *N*‐glycans, which had previously been implicated in electrophysiological changes that determine the fate potential of neural stem cells, whereby highly branched *N*‐glycans on neural stem and progenitor cells significantly increased the *C*_eff_ leading to the generation of more astrocytes at the expense of neurons [41]. The cyclic *C*_eff_ may also be associated with lipid species with lipidomics analysis of human plasma and skeletal muscle which revealed that 13% of all detected lipids displayed significant day–night rhythmicity. The most rhythmic lipids were glycerophospholipids and diacylglycerols, with the latter being the largest fraction (>50% of all rhythmic species) [42, 43, 44]. Looking at the cyclic expression of *C*_eff_ and *G*_eff_ collectively, it may be attributed to SWELL1 (also called Leucine–Rich Repeat Containing Protein 8a, or LRRC8a) – a regulator of adipocyte size [45] and essential component of the volume‐regulated anion channel, with ion‐conductive signalling properties [46]. These speculative notions require further investigation to warrant any correlation.

The other major difference between the RBC rhythms reported in the literature and those observed here is in *G*_eff_. In human RBCs, *G*_eff_ was observed to exhibit a circadian (∼24 h) rhythm, in counterphase to the *σ*_cyto_ [18]. In pre‐adipocytes, we observe an *ultradian* rhythm in *G*_eff_ with no corresponding rhythm in *σ*_cyto_. Ultradian rhythms are present in many biological systems, including gene expression in mice *in vivo* and *in vitro* [47, 48]. In RBCs, circadian changes in *G*_eff_ and *σ*_cyto_ were associated with changes in rhythmic potassium transport regulating the circadian clock in these cells [18]. The authors also suggested that a rhythmic regulation of transmembrane ion transport in RBCs may contribute to, or at least influence, mechanism(s) determining the period of oscillation. Thus far, a rhythm in the abundance of peroxiredoxin, highly conserved antioxidant proteins, has served as the primary reporter for circadian timekeeping in RBCs (which lack nuclei and TTFL based circadian clocks) [4]. These rhythms were temperature entrained and compensated, both key features of circadian rhythms [4, 36].

Peroxiredoxin circadian rhythms were also found in murine NIH3T3 fibroblasts and mouse embryonic fibroblasts [4]. Additionally, circadian cycles of posttranslational modification of peroxiredoxin have previously been reported in murine liver [4]. Moreover, circadian regulation of transmembrane ion transport, whose initial discovery predates the identification of clock genes [49, 50, 51], has become the subject of renewed interest in light of observations that cellular Mg^2+^ and K^+^ transports exhibit circadian rhythms in several mammalian cell types, as well as in algae and fungal cells, suggestive of a circadian function with greater evolutionary conservation than any known clock gene [52, 53]. Now that we have identified these rhythms, an unsynchronised time‐series could indicate that it is driven by a serum synchronised clock (as the TTFL is).

## 5 CONCLUDING REMARKS

In summary, we have demonstrated that circadian rhythms are readily identified and quantified *in vitro* pre‐adipocytes using DEP. The cells demonstrated robust but phase‐shifted circadian rhythms in radius and membrane capacitance; as well as near 12 h, ultradian rhythms in membrane conductance and no rhythm in cytoplasmic conductivity. These point to different underlying mechanisms to those observed in human RBCs and underline DEP as a potential tool for unpicking the role of electrical behaviour in cellular clock mechanisms in nucleated cells in the future. RBCs are a specific and unusual type of cells; they are highly regular, anucleate, and possess some DEP properties (such as a strong dependence of *G*_eff_ and *C*_eff_ on the medium conductivity) which are not observed in other cell types. As a consequence, this demonstration of circadian rhythms in a nucleated, adherent cell line represents a significant step forward in DEP analysis of circadian rhythms, as it demonstrates that these DEP rhythms are in fact generalisable across a wide range of mammalian cells.

## CONFLICT OF INTEREST

The authors have declared no conflict of interest.

## Data Availability

The data that support the findings of this study are available from the corresponding author upon reasonable request.
